# Supplementary material for: Regulation of human trophoblast gene expression by endogenous retroviruses
Source: Nat Struct Mol Biol. 2023 Apr 3;30(4):527–38. doi: 10.1038/s41594-023-00960-6 (PMC10113160; doi:10.1038/s41594-023-00960-6)
Supplement: Supplementary file 1 — Reporting Summary [file 41594_2023_960_MOESM1_ESM.pdf]

nature portfolio

Corresponding author(s): Miguel Branco

Last updated by author(s): Feb 3, 2023

Reporting Summary

Nature Portfolio wishes to improve the reproducibility of the work that we publish. This form provides structure for consistency and transparency in reporting. For further information on Nature Portfolio policies, see our Editorial Policies and the Editorial Policy Checklist.

Statistics

For all statistical analyses, confirm that the following items are present in the figure legend, table legend, main text, or Methods section.

n/a	Confirmed	
☐	☒	The exact sample size (*n*) for each experimental group/condition, given as a discrete number and unit of measurement
☐	☒	A statement on whether measurements were taken from distinct samples or whether the same sample was measured repeatedly
☐	☒	The statistical test(s) used AND whether they are one- or two-sided<br>*Only common tests should be described solely by name; describe more complex techniques in the Methods section.*
☒	☐	A description of all covariates tested
☐	☒	A description of any assumptions or corrections, such as tests of normality and adjustment for multiple comparisons
☐	☒	A full description of the statistical parameters including central tendency (e.g. means) or other basic estimates (e.g. regression coefficient) AND variation (e.g. standard deviation) or associated estimates of uncertainty (e.g. confidence intervals)
☐	☒	For null hypothesis testing, the test statistic (e.g. *F*, *t*, *r*) with confidence intervals, effect sizes, degrees of freedom and *P* value noted<br>*Give P values as exact values whenever suitable.*
☒	☐	For Bayesian analysis, information on the choice of priors and Markov chain Monte Carlo settings
☒	☐	For hierarchical and complex designs, identification of the appropriate level for tests and full reporting of outcomes
☒	☐	Estimates of effect sizes (e.g. Cohen's *d*, Pearson's *r*), indicating how they were calculated

Our web collection on statistics for biologists contains articles on many of the points above.

Software and code

Policy information about availability of computer code

Data collection	No software was used for data collection.
Data analysis	Open source software: trim galore, Bowtie2 v2.1.0, MACS2 v2.1.1 , SEACR v1.2 , Hisat2 v2.0.5 , DESeq2 v1.36.0, deepTools2.0, StringTie v1.3.3b, MEME SUITE v5.0.1, Seqmonk v1.47.2, SQuIRE v0.9.9.9a-beta. Custom scripts used for data analysis are available at https://github.com/MBrancoLab/Frost_2022_hTroph.

For manuscripts utilizing custom algorithms or software that are central to the research but not yet described in published literature, software must be made available to editors and reviewers. We strongly encourage code deposition in a community repository (e.g. GitHub). See the Nature Portfolio guidelines for submitting code & software for further information.

Data

Policy information about availability of data

All manuscripts must include a data availability statement. This statement should provide the following information, where applicable:

- Accession codes, unique identifiers, or web links for publicly available datasets
- A description of any restrictions on data availability
- For clinical datasets or third party data, please ensure that the statement adheres to our policy

CUT&Tag, CUT&RUN, ChIP-seq and RNA-seq data have been deposited in NCBI's Gene Expression Omnibus under accession number GSE200763.
Details of other datasets used can be found in Supplementary Table S7.
Genome assemblies used: hg38, mm10, calJac4, gorGor6, micMur2, nomLeu3, panTro6, ponAbe3, rheMac10, tarSyr2.
The Github repository https://github.com/MBrancoLab/Frost_2022_hTroph contains the following source data: 1) TE family enrichments for chromatin features ('Peak_enrichment' folder), 2) motif frequencies ('Transcription_factors/FIMO'), 3) JUN/JUND binding profiles ('Transcription_factors/AP1_profiles'), 4) processed

RNA-seq data ('RNA-seq' and 'Comparative_analysis'), 5) TE orthology ('Active_families/orthologues'), 6) RT-qPCR, growth curves, FACS and ELISA ('Assays'). A Readme file is included describing how each figure was generated.

Field-specific reporting

Please select the one below that is the best fit for your research. If you are not sure, read the appropriate sections before making your selection.

☒ Life sciences ☐ Behavioural & social sciences ☐ Ecological, evolutionary & environmental sciences

For a reference copy of the document with all sections, see nature.com/documents/nr-reporting-summary-flat.pdf

Life sciences study design

All studies must disclose on these points even when the disclosure is negative.

Sample size	Sample sizes were not statistically predetermined, but were based on similar studies. TE-derived enhancers were derived from two replicates in hTSCs, with similar results in another two independent datasets (see 'Replication' below). RNA-seq of SP600125-treated cells was performed in triplicate and sufficiently powered to detect 10% differences at p<0.05. The effects of CRISPR experiments were performed in variable numbers of replicates (see details in manuscript) depending on the assay and observed variability; effect sizes of ~2-fold could be robustly detected.
Data exclusions	No data were excluded from the analyses.
Replication	TE-derived enhancers in human trophoblast were derived from two hTSC CUT&Tag runs, one hTSC ChIP-seq and two published ChIP-seq sets from primary trophoblast, with similar results. All results from RNA-seq data analyses were successfully replicated between hTSCs and primary cytotrophoblast. Results from CRISPR experiments were successfully replicated by performing independent infections and/or using different sgRNA pairs (as detailed in the main text and figure legend).
Randomization	This study used cell lines, for which randomization is not applicable. When treatments were used (CRISPSR, JNK inhibition), the same cell population was split into two or more equal subpopulations.
Blinding	Blinding was used for manually quantifying cell invasion assays. All other data analyses were independent of subjective human-based decisions, using objective and standardised data analysis pipelines, and therefore blinding wasn't used.

Reporting for specific materials, systems and methods

We require information from authors about some types of materials, experimental systems and methods used in many studies. Here, indicate whether each material, system or method listed is relevant to your study. If you are not sure if a list item applies to your research, read the appropriate section before selecting a response.

Materials & experimental systems

n/a	Involved in the study
☐	☒ Antibodies
☐	☒ Eukaryotic cell lines
☒	☐ Palaeontology and archaeology
☒	☐ Animals and other organisms
☒	☐ Human research participants
☒	☐ Clinical data
☒	☐ Dual use research of concern

Methods

n/a	Involved in the study
☐	☒ ChIP-seq
☒	☐ Flow cytometry
☒	☐ MRI-based neuroimaging

Antibodies

Antibodies used

HLAG (APC anti-human HLA-G Antibody Clone: 87G, BioLegend UK #335905)
H3K4me3 (RRID:AB_2616052, Diagenode C15410003)
H3K4me1 (RRID:AB_306847, Abcam ab8895)
H3K27ac (RRID:AB_2637079, Diagenode C15410196)
H3K27Ac (39034, Active Motif)
H3K9me3 (C15410193, Diagenode)
H3K27me3 (C15410195, Diagenode)
c-Jun (60A8 – 9165T, Cell Signalling)
JunD (D17G2 – 5000S, Cell Signalling)
GATA3 (sc-268, Santa Cruz)
TFAP2C (sc-12762, Santa Cruz)
TEAD4 (CSB-PA618010LA01HU, Stratech)
rabbit IgG (sc-2027, Santa Cruz)
Guinea Pig anti-Rabbit IgG (ABIN101961, Antibodies-Online)

pSer63 c-Jun (#2361, Cell Signalling)
α-Tubulin (T9026, Sigma Aldrich)
H3 (ab1791, Abcam)
peroxidase conjugated anti-rabbit IgG (A6154, Sigma Aldrich)
peroxidase conjugated anti-mouse IgG (A0168, Sigma Aldrich)
SDC-1/CD138 (clone MI15, Fisher Scientific #15892669)
Goat anti-Mouse IgG, Alexa Fluor Plus 488 (Catalog # A32723, Thermo Fisher Scientific)

Validation

HLAG - clone 87G has been validated by HLA-G overexpression in PMID:34201301, and used for IF and flow cytometry.
ChIP-grade antibodies from Diagenode (H3K4me3, H3K27ac, H3K9me3, H3K27me3) were extensively validated by the manufacturer using dot blots, western blots, ChIP-seq, and others.
H3K4me1 (Abcam ab8895, ChIP-grade) - validated by western blot and ChIP-seq (manufacturer's website).
H3K27ac (Active Motif 39034) - validated by the manufacturer for ChIP-seq (and similar), western blot and IF.
c-Jun (60A8 – 9165T, Cell Signalling) - validated for western blot using c-Jun knockout HeLa cells (manufacturer's website).
JunD (D17G2 – 5000S, Cell Signalling) - validated in PMID:35926467 by JunD knockdown.
Santa Cruz antibodies (GATA3, TFAP2C) were validated by overexpression of the respective protein (manufacturer's website).
TEAD4 (CSB-PA618010LA01HU, Stratech) - validated by western blot (Cusabio website).
pSer63 c-Jun (#2361, Cell Signalling) - validated by activation of c-Jun kinases using anisomycin (manufacturer's website).
α-Tubulin (T9026, Sigma Aldrich) - validated by western blot and IF (manufacturer's website).
H3 (ab1791, Abcam) - validated by the manufacturer by blocking the antibody with an H3 peptide.
SDC-1/CD138 (clone MI15) - validated in PMID:10027728 using recombinant proteins.

Eukaryotic cell lines

Policy information about cell lines

Cell line source(s)

hTSCs were obtained from the RIKEN cell bank (RIKEN BioResource Research Center, 305-0074 Japan).
HEK293T cells were a gift from Prof. Silvia Marino (QMUL)

Authentication

hTSCs were validated by qRT-PCR of marker genes, differentiation into ST and EVT.
No validation was performed on 293T cells, as they were used just to produce lentiviruses.

Mycoplasma contamination

Cells tested negative for mycoplasma

Commonly misidentified lines
(See ICLAC register)

No commonly misidentified cell lines were used.

ChIP-seq

Data deposition

☒ Confirm that both raw and final processed data have been deposited in a public database such as GEO.

☒ Confirm that you have deposited or provided access to graph files (e.g. BED files) for the called peaks.

Data access links
May remain private before publication.

https://www.ncbi.nlm.nih.gov/geo/query/acc.cgi?acc=GSE200763

Files in database submission

Raw fastq files, peak files, bigwig tracks.

Genome browser session
(e.g. UCSC)

Human: http://epigenomegateway.wustl.edu/browser/?sessionFile=https://data.cyverse.org/dav-anon/iplant/home/mbranco/hTroph/eg-session-lV_cZJGeY-ec032020-d6e7-11ec-9604-a1ccb72a2b23.json
Mouse: http://epigenomegateway.wustl.edu/browser/?sessionFile=https://data.cyverse.org/dav-anon/iplant/home/mbranco/mTroph/eg-session--41d93570-d6ed-11ec-a762-1750142ba3bb.json

Methodology

Replicates

hTSC and mTSC H3K27ac, H3K4me1 and H3K4me3 CUT&Tag were performed in duplicate. All other experiments are single replicates.

Sequencing depth

Sample:	Read count:
hTSC H3K27ac ChIP	40,213,296
hTSC H3K4me1 ChIP	79,194,135
hTSC H3K4me3 ChIP	61,449,294
hTSC Input ChIP	83,823,113
hTSC H3K27ac CUT&Tag 1	7,871,525
hTSC H3K27ac CUT&Tag 2	4,859,297
hTSC H3K4me1 CUT&Tag 1	8,861,507
hTSC H3K4me1 CUT&Tag 2	12,678,027
hTSC H3K4me3 CUT&Tag 1	7,867,045
hTSC H3K4me3 CUT&Tag 2	19,393,419
hTSC cJun CUT&Tag	5,652,603
hTSC JunD CUT&Tag	5,864,304
hTSC IgG CUT&Tag	2,486,061

hTSC H3K9me3 CUT&Tag	19,549,401
hTSC H3K27me3 CUT&Tag	6,095,557
hTSC GATA3 CUT&RUN	3,194,528
hTSC TEAD4 CUT&RUN	21,992,284
hTSC TFAP2C CUT&RUN	5,407,256
hTSC IgG CUT&RUN	3,538,560
EVT H3K27ac CUT&Tag	3,892,494
EVT IgG CUT&Tag	1,226,230
mTSC H3K27ac CUT&Tag 1	16,719,388
mTSC H3K27ac CUT&Tag 2	9,610,089
mTSC H3K4me1 CUT&Tag 1	15,974,036
mTSC H3K4me1 CUT&Tag 2	16,650,467
mTSC H3K4me3 CUT&Tag 1	16,803,789
mTSC H3K4me3 CUT&Tag 2	10,955,327
mTSC IgG CUT&Tag 1	4,583,747
mTSC IgG CUT&Tag 2	567,885

Antibodies

H3K4me3 (RRID:AB_2616052, Diagenode C15410003)
H3K4me1 (RRID:AB_306847, Abcam ab8895)
H3K27ac (RRID:AB_2637079, Diagenode C15410196)
H3K27Ac (39034, Active Motif)
H3K9me3 (C15410193, Diagenode)
H3K27me3 (C15410195, Diagenode)
c-Jun (60A8 – 9165T, Cell Signalling)
JunD (D17G2 – 5000S, Cell Signalling)
GATA3 (sc-268, Santa Cruz)
TFAP2C (sc-12762, Santa Cruz)
TEAD4 (CSB-PA618010LA01HU, Stratech)
rabbit IgG (sc-2027, Santa Cruz)
Guinea Pig anti-Rabbit IgG (ABIN101961)

Peak calling parameters

ChIP-seq peaks were called using MACS2 v2.1.1 with -q 0.05 and --broad.
CUT&Tag and CUT&RUN peaks were called using SEACR v1.2 with normalisation to IgG and relaxed mode.

Data quality

FastQC was used to assess raw data quality. Peak detection was performed with a q-value cut-off of 0.05.

Software

MACS2 v2.1.1 and SEACR v1.2 for peak detection. Other custom scripts are available at https://github.com/MBrancoLab/Frost_2022_hTroph.
