# Supplementary figures and images for: Regulation of human trophoblast gene expression by endogenous retroviruses
Source: Nat Struct Mol Biol. 2023 Apr 3;30(4):527–38. doi: 10.1038/s41594-023-00960-6 (PMC10113160; doi:10.1038/s41594-023-00960-6)

Phosphorylated c-Jun

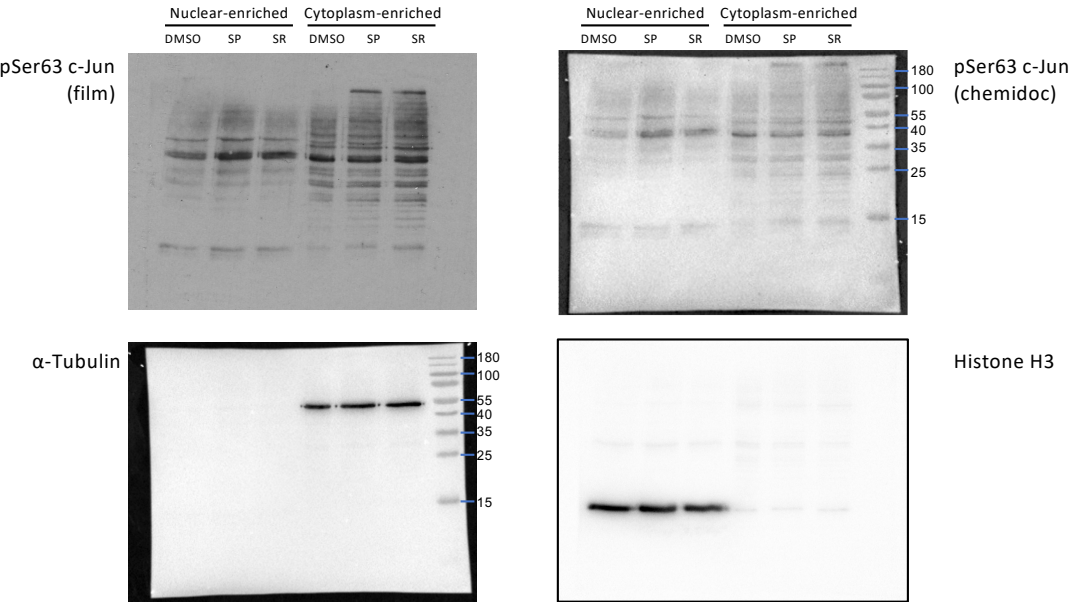

Total c-Jun

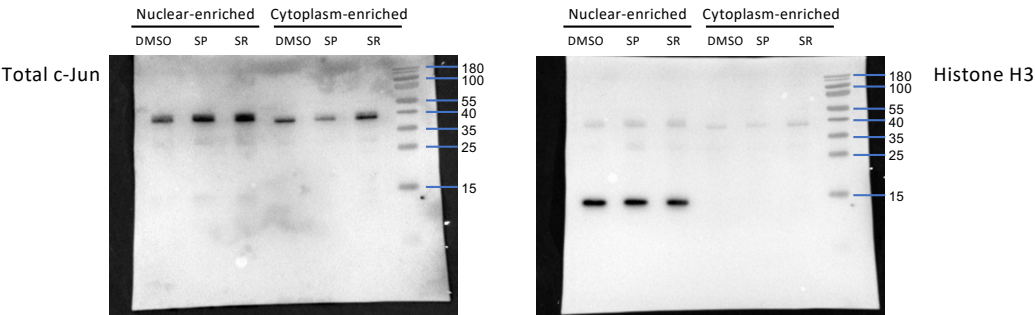

Supplement: Extended Data Fig. 4 — Unprocessed western blots. [file 41594_2023_960_MOESM4_ESM.pdf]
